# The development of type 2 diabetes amongst sibling pairs in the South‐South region of Nigeria: The effect of genetics, environment and lifestyle

**DOI:** 10.1111/dme.70278

**Published:** 2026-04-01

**Authors:** Sunny Chinenye, Ibi Erekosima, Adrian H. Heald, Gesiye Bozimo, Simon G. Anderson, Samuel Seidu, Kariba Akhidue, Adaeze Ugwu, Chioma Unachukwu, Ibitrokoemi Korubo, Kingsley Chukwuemeka Patrick‐Iwuanyanwu, Victoria Gomba, Uwoma Batubo, Victoria Akanatei, Victor Ken‐Uba, Iyeopu Siminialayi, Sokiprim Akoko, Justin Ideozu, Hamilton Opurum

**Affiliations:** ^1^ Department of Internal Medicine University of Port Harcourt Port Harcourt Nigeria; ^2^ Department of Internal Medicine University of Port Harcourt Teaching Hospital Port Harcourt Nigeria; ^3^ Department of Internal Medicine University of Manchester Manchester UK; ^4^ Department of Internal Medicine Salford Royal Hospital Salford UK; ^5^ Department of Internal Medicine Federal Medical Centre Yenagoa Nigeria; ^6^ Department of Internal Medicine Niger Delta University Teaching Hospital Okolobiri Nigeria; ^7^ Caribbean Institute of Health Research The University of the West Indies Cave Hill Campus Barbados; ^8^ Leicester Real World Evidence Unit Leicester Diabetes Centre Leicester UK; ^9^ Leicester Diabetes Research Centre University Hospital Leicester, Leicester General Hospital Leicester UK; ^10^ Rivers State University Teaching Hospital Port Harcourt Nigeria; ^11^ Department of Pharmacology University of Port Harcourt Port Harcourt Nigeria; ^12^ MYAFRODNA LTD Biobank & Biorepository Port Harcourt Nigeria; ^13^ Department of Medical Biochemistry University of Port Harcourt Port Harcourt Nigeria

**Keywords:** environment, genetics, sib‐pairs, South–South Nigeria, type 2 diabetes

## Abstract

**Introduction:**

Type 2 diabetes is a complex condition with a multifactorial pathogenesis. The pathogenesis is considered to be polygenic due to complex interactions between genetic/epigenetic and environmental factors.

The aim of this study is to determine the genetic, environmental and lifestyle factors associated with the development of type 2 diabetes amongst sibling pairs (sib‐pairs) in the South‐South region of Nigeria and determine which is more prominent.

**Methods:**

This multicentre cross‐sectional, comparative exploratory study will recruit 250 sib‐pairs living with type 2 diabetes using a simple consecutive sampling method. Participants will be assessed for bio‐psycho‐social and environmental risk factors that may put them at risk of developing type 2 diabetes using a structured survey instrument. Blood samples collected for multi‐omic studies will be analysed for genetic and environmental factors associated with the development of type 2 diabetes.

**Results:**

It is expected that the most common genetic factors associated with the development of type 2 diabetes in sib‐pairs will be explored and it will be determined to what extent they are responsible for the development of type 2 diabetes when compared to lifestyle and environmental factors.

**Conclusion:**

While there is a strong genetic component to the development of type 2 diabetes, it is often triggered by lifestyle or environmental factors. Understanding the genetics that lead to the development of type 2 diabetes in sib‐pairs when compared to the inherent environmental and lifestyle risk factors may lead to the development of new therapeutic and preventive strategies.

**What's new?**

- This multicentre cross‐sectional, comparative exploratory study will recruit 250 sib‐pairs living with type 2 diabetes. The most common genetic factors associated with the development of type 2 diabetes in sib‐pairs will be explored.
- While there is a strong genetic component to the development of type 2 diabetes, it is often triggered by lifestyle or environmental factors.
- Understanding the genetics that lead to the development of type 2 diabetes in sib‐pairs when compared to the environmental and lifestyle risk factors may lead to the possible development of new therapeutic and preventive strategies.

## 1 INTRODUCTION

Diabetes mellitus (DM) is a major public health problem with growing concern. The predominant form globally is type 2 diabetes, which accounts for about 95% of cases.^1^ The International Diabetes Federation (IDF) estimated in 2024 that 588.7 million people worldwide had type 2 diabetes, with an expected rise to 852.5 million people by 2050.^1^ 24.6 million adults aged 20–79 years were estimated to have diabetes in Africa, while Nigeria was estimated to have 1.7 million citizens living with diabetes in 2024.^1^, ^2^ The South‐South geopolitical zone of Nigeria has the highest prevalence (9.8%) of diabetes among the six geopolitical zones of Nigeria.^3^

Genetics and family history have been shown to have a very strong link to the development of not just type 1, but also type 2 diabetes.^4^ Recent genome‐wide association studies (GWAS) have added at least 83 genetic variants to the list of already well characterized risk predictors of type 2 diabetes, including family history and obesity.^4^, ^5^ Although these variants can significantly predict type 2 diabetes either alone or as part of genotype risk scores, they do not yet offer clinical discrimination beyond that achieved with common clinical measurements. Further research may improve the predictive performance of genotype information. Expanded GWAS efforts in non‐European populations will allow targeted sequencing of risk loci and the identification of true causal variants. Also, studies with longer prediction time horizons may demonstrate that genotype information performs better than clinical risk predictors over a longer period.^6^

Hyperglycaemia induces epigenetic changes by increasing the production of inflammatory proteins through its action on the NFkB p65 promoter unit.^7^ Current studies also show that insulin resistance in type 2 diabetes is largely dependent on epigenetic, environmental, dietary and lifestyle factors such as physical inactivity, unhealthy diet and stress.^8^

Environmental factors like air pollution change endothelial function and increase Insulin resistance.^8^ The South‐South region of Nigeria including Port Harcourt, and some regions of Bayelsa state has a high exposure to environmental degradation due to oil drilling and gas flaring and this results in the release of particulate matter including particulate matter_2.5_ (P.M_2.5_) and _0.1_.^9^

Long‐term exposure to particulate matter leads to vascular inflammation, insulin resistance and endothelial dysfunction thus increasing the risk of cardiovascular disease and type 2 diabetes.^1^, ^8^ One‐fifth of the global burden of type 2 diabetes may be due to PM_2.5_ pollution.^10^ The PM_2.5_ concentration in Port Harcourt is often several times higher than the WHO annual air quality guideline value and this has a significant effect on public health and may also contribute to the development of type 2 diabetes in the region.^9^, ^10^, ^11^ Other environmental factors associated with type 2 diabetes include stress, sleep deprivation and exposure to environmental toxins including pesticides.^12^

Lifestyle factors associated with developing type 2 diabetes include sedentary lifestyles, unhealthy eating habits and being overweight or obese.^1^, ^8^

Family history and genetics play a significant role in the development of type 2 diabetes. In identical twins, for example, when one twin has type 1 diabetes, the other one has a 50% chance of developing it, but in type 2 diabetes, the risk of the other twin developing it is as high as 75%.^13^, ^14^, ^15^

In type 2 diabetes, the ‘*diabetes genes*’ may exhibit only subtle variation in the gene sequence, and these variations may be extremely common. The difficulty lies in linking such common gene variations, known as single nucleotide polymorphisms (SNPs), with an increased risk of developing diabetes.^16^

Some genes that have been found to be associated with the risk of developing type 2 diabetes mellitus include: TCF7L2, ABCC8, CAPN10, GLUT2, GCGR, APOAII, FTO, PON1, ADIPOQ, MTHFR, PPARG, G6PC2, GCK, GCKR, OCT3 and HNF4A.^17^, ^18^, ^19^

One method of finding diabetes susceptibility genes is through whole‐genome linkage studies in which the entire genome of affected family members is scanned, and the families are followed over several generations with large numbers of affected sib‐pairs being studied.^18^, ^19^

Genetic variants that modulate insulin action may have an increased effect on type 2 diabetes susceptibility in the presence of obesity, while genetic variants acting on insulin secretion may have a greater impact on type 2 diabetes susceptibility in non‐obese individuals.^17^, ^18^, ^19^

Findings from genome‐wide association and exome chip studies suggest the presence of both ancestry‐specific and shared loci for type 2 diabetes. Among the European‐identified loci that are transferable to individuals of African ancestry, allelic heterogeneity as well as differential linkage disequilibrium and risk allele frequencies pose challenges and opportunities for fine mapping and identification of causal variant(s) by trans‐ancestry meta‐analysis.^19^, ^20^

About 100 polymorphisms in 57 genes have been investigated in relation to type 2 diabetes in populations within Africa, in 60 studies. Almost all studies used the candidate gene approach, with >88% published during 2006–2014 and 70% (42/60) originating from Tunisia and Egypt. Polymorphisms in ACE, AGRP, eNOS, GSTP1, HSP70‐2, MC4R, MTHFR, PHLPP, POL1, TCF7L2 and TNF‐α gene were found to be associated with type 2 diabetes, with overlapping effect on various cardiometabolic traits. The polymorphisms investigated in multiple studies mostly had consistent effects across studies, with only modest or no statistical heterogeneity.^21^, ^22^

Some studies carried out in Sub‐Saharan Africa on the characteristics and development of type 2 diabetes showed a preference for maternal aggregation, a higher prevalence among first‐degree compared to second‐degree relatives, early age‐onset (<50 years) and inherited abnormalities of beta‐cell function/mass. The overall prevalence of type 2 diabetes was 28.2% for the population with a positive family history and 11.2% for the population with a negative family history.^22^, ^23^, ^24^, ^25^

The risk of type 2 diabetes is approximately 2‐fold higher in African Americans than in European Americans, even after adjusting for known environmental risk factors, including socioeconomic status (SES), suggesting that genetic factors may explain some of this population difference in disease risk.^26^ However, relatively few genetic studies have examined this hypothesis in non‐European or African populations. Most current evidence on the genetic markers of type 2 diabetes in African populations mostly originates from North African countries and are largely insufficient to reliably inform the genetic architecture of type 2 diabetes across Africa.^27^

The consensus is that more genetic research is needed in African ancestry populations, including next‐generation sequencing, to improve the understanding of the genetic architecture of type 2 Diabetes. In indigenous African populations, there is also the added factor of polygamy, which is very common, and as such, siblings may be fully or partially genetically related and this may have an effect on the expression of the relevant genes.

The South‐South geopolitical zone of Nigeria is said to have the highest prevalence of type 2 diabetes in Nigeria and thus may be expected to have the highest burden of complications.^2^, ^3^ The early identification of the factors that may play the largest role in the development of type 2 diabetes may help to reduce the prevalence and slow the progression of the disease. Early interventions can also be instituted in order to reduce the risk of possible complications.

## 2 OBJECTIVE

To compare the environmental, lifestyle and genetic risk factors associated with type 2 diabetes among sib‐pairs affected with type 2 diabetes in the South‐South region of Nigeria and determine which of them is more predominant.

## 3 MATERIALS AND METHODS

### 3.1 Project sites/locations

The study will be a multi‐site study involving the Medical Outpatient Clinics of 4 tertiary hospitals: The University of Port Harcourt Teaching Hospital (UPTH), Rivers State University Teaching Hospital (RSUTH), Federal Medical Centre Yenagoa (FMCY) and Niger Delta University Teaching Hospital (NDUTH). These four hospitals are the major tertiary hospitals in Rivers and Bayelsa states and also serve as referral centres for the neighbouring South‐South states.

Each site will have a Principal Investigator and a complement of staff who will carry out the study. The coordinating site will be the University of Port Harcourt Teaching Hospital. A Favourable Ethical Opinion for the project has been given by the ethics board at Port Harcourt Teaching Hospital. Reference 2025/33.

### 3.2 Project population

The study population for this initial, exploratory study will be drawn from 250 adult sib‐pairs of people living with type 2 diabetes, that is one patient living with type 2 diabetes who is attending one of the Medical Outpatient Clinics of a participating site and 1 sibling with type 2 diabetes. In a case where a patient is a twin, such a sib‐pair will be given priority in recruitment. Five hundred apparently healthy, randomly selected controls (without diabetes) matched for age and gender to the study participants will also be recruited. A total of 1000 participants are thus expected to be recruited for the study.

### 3.3 Inclusion/exclusion criteria

The Inclusion criteria for participants who will be included in the study include:

1. Participants with type 2 diabetes aged between 18 and 75 years.
2. Participants with type 2 diabetes who have been identified as having at least 1 sibling with type 2 diabetes who is over the age of 18 years.
3. Siblings of participants with type 2 diabetes aged 18 years and above who give consent to participate in the study.
4. Participants with type 2 diabetes who live in the target area.
5. Participants with diabetes or their siblings who have learning disabilities but are able to give informed consent to participate in the study.

The exclusion criteria will include:

1. Participants with type 1 diabetes.
2. All participants with type 2 diabetes who are younger than 18 years of age at the time of the study.
3. Participants who are older than 75 years of age at the time of the study.
4. Women participants who are pregnant at the time of the study.

### 3.4 Study instrument

The study instrument that will be used is a structured, interviewer‐administered questionnaire (Appendix A) that will explore the sociodemographic data of participants, including gender, age and ethnicity. The risk factors for type 2 diabetes, including a family history of type 2 diabetes, the history and quantity of exercise done, exposure to environmental toxins, air and water pollution, as well as dietary history, shall be looked at. The duration of diabetes since diagnosis, medications taken, adherence to medication, presence of comorbidities and health‐seeking behaviour shall also be explored. Specific family relationships will be explored to determine the exact nature of the sib‐pair relationships. Psychological evaluations and details on sleep history and food security will be assessed.

Anthropometric measurements of Height, Weight, Body Mass Index (BMI), Waist circumference (WC), Neck circumference (NC) and Hip circumference (HC) will be done. The pulse rate and Blood Pressure (BP) and heart rate variability will be measured.

At phlebotomy, blood will be collected in sodium (Na) heparin, EDTA, Streck and PAXgene tubes, processed and stored for serum testosterone, serum electrolytes, urea and creatinine, plasma insulin, serum C‐peptide, sex human binding globulin (SHBG), fasting blood glucose (FBG), fasting lipid profile (FLP), glycosylated haemoglobin (HbA1c), genotype, high sensitivity CRP, vitamin A, vitamin D, vitamin E, folate, serum iron and serum calcium measurements. Urinary albumin/creatine excretion ratio will be determined.

DNA extraction from whole blood quantification and sequencing of the most relevant genetic markers that are associated with type 2 diabetes will be done.

Each participant in the study will spend approximately 1 h answering the questionnaires and having urine and blood samples taken from them.

### 3.5 Study procedure

Advocacy for the study will be done using the Diabetes Association of Nigeria (DAN) patient groups at each site to create awareness about the study and recruit willing participants. Recruited participants will be adequately counselled on the nature of the study and the procedures involved. After written, informed consent has been obtained, a total of 20 mLs of blood will be drawn from each participant: 4 mL of whole blood will be taken for the genetic studies and stored in an Ethylenediaminetetraacetic acid (EDTA) tube and stored at –80°C. The rest of the blood sample will be divided into aliquots of 2 mL each of serum and stored in lithium‐heparin tubes and used for the other investigations including serum electrolytes, urea, creatine, serum lipids, C‐peptide, sex hormone binding globulin (SHBG), genotype, high sensitivity CRP, vitamin A, vitamin D, vitamin E and folate levels. Investigations that are not routinely done shall be batched for future analysis. The blood samples for the genetic and future serological studies will be stored at −80°C while the other blood tests will be done on the same day they are collected.

### 3.6 Genetic analysis

DNA will be extracted from a whole blood sample and specific gene variants, for example TCF7L2, will be identified using Polymerase Chain Reaction (PCR) for allele‐specific primers. The DNA will then be amplified using Polymerase Restriction Fragment Length Polymorphism (PCR‐RFLP) techniques, and single nucleotide polymorphisms (SNPs) genotyping shall be done.

RNA will be extracted for gene expression and quantified by quantitative polymerase chain reaction (qPCR) techniques.

For blood samples collected directly into PAXgene™ blood RNA tubes, the total cellular RNA will be extracted from the samples with the PAXgene™ blood RNA kit (Qiagen) according to the manufacturer's protocol, 24 h or 5 days after phlebotomy.

All investigations apart from the genetic studies will be done in a designated laboratory in Port Harcourt, Rivers state while the blood for the genetic studies will be transported using best standard practices to a laboratory outside Nigeria for analysis. However, before sample collection commences, if an International Organization for Standardization (ISO) certified laboratory in Nigeria is identified and is able to conduct the genetic testing, all genomic investigations shall be done in Nigeria.

The data and samples for the study will be collected by the Investigators and research assistants (RAs) who will be drawn from among other health care personnel in the participating hospitals with the requisite medical training. There will be no risk to either the participants or researchers and this will be clearly explained to the study participants. All concerns or queries will be addressed to the Principal Investigator at each hospital, who will adequately address all such concerns.

### 3.7 Data management

The data collection and storage will be done using the secure web application research Electronic Data Capture (REDCap), on a HIPAA‐compliant server, while software that is specific for genetic analysis will be used. The study will adhere to the STROBE guidelines for observational research, data collection and reporting.

## 4 ETHICAL CONSIDERATIONS

Local approval was obtained from the Research and Innovation committees of each participating hospital. Participants will give informed consent before recruitment into the study, and those who do not give consent to be recruited as participants shall not be denied medical care as a result. All data obtained will be anonymized and participants with identified abnormalities from immediately available investigation results will be counselled and referred for proper treatment and follow‐up.

## 5 STRENGTHS AND LIMITATIONS OF THE STUDY

Strengths of the study are that this investigation will help to predict future disease risk in African‐origin individuals. The study will be easily reproducible as the methodology is clear.

Limitations are that the study is resource‐intensive and this may hinder the undertaking of the study on a larger scale in Nigeria or elsewhere in Africa.

## 6 CONCLUSION

While there is a strong genetic component to the development of type 2 diabetes, it is often triggered by lifestyle or environmental factors. Understanding the genetics that lead to the development of type 2 diabetes in sib‐pairs when compared to the environmental and lifestyle risk factors may lead to the possible development of new therapeutic and preventive strategies.

## PROJECT DURATION

The study is expected to be carried out over a period of 12–18 months.

## AUTHOR CONTRIBUTIONS

All the authors contributed to the conceptualization and execution of the paper and approved the final version being submitted.

## FUNDING INFORMATION

This research has not received any specific grant from funding agencies in the public, commercial, or not‐for‐profit sectors.

## CONFLICT OF INTEREST STATEMENT

The authors declare that they have no conflict of interest at this time.

## APPENDIX A STUDY QUESTIONAIRE

**Section A: Bio‐data**

1. Interviewer code ………………………………………….
2. Participant Study Identification…………………………….
3. Phone number…………………………………………….
4. DOB (DD/MM/YYYY)………………………………….
5. Age (in years)…………………………………………….
6. Sex a. Male………….b. Female……………………
7. Address…………………………………………………………………………….

**Section B: Socio‐demographics**

1. **Marital status** (circle one)
  - Single
  - Married
  - Separated
  - Divorced
  - Widow/widower
2. **Nationality**
  - Nigerian
  - Others................
3. **State of origin** (If Nigerian):............
4. **Tribe** (circle one)
  - Ikwerre.
  - Kalabari
  - Ogoni
  - Ibani
  - Ekpeye
  - Igbo
  - Yoruba
  - Hausa
  - Other (please specify)…………………….
5. **Occupation** (circle one)
  - Employed Professional (doctor, lawyer, engineer, etc.)
  - Managerial and lower professionals (managers, teachers)
  - Non‐manual skilled occupations (office workers)
  - Manual skilled occupations (bricklayers, coalminers, traders).
  - Semi‐skilled occupations (e.g. postal workers)
  - Unskilled occupations (e.g. security guard, porter, farmer)
  - Unemployed
  - Students
6. **Educational status** (circle one)
  - No formal education
  - Some primary schools.
  - Completed primary school.
  - Some secondary school
  - Completed secondary school
  - Higher education (below degree level—e.g. OND).
  - Higher education (Degree level—HND/Bachelor's degree)
  - Postgraduate
7. **Religion**
  - Christian
  - Muslim
  - Traditional
  - Others

**Section C: Health behaviour**

8 **Tobacco use**
  - Do you currently smoke any tobacco products, such as cigarettes, cigars or pipes, shisha?
    - Yes
    - No
  - If yes, do you currently smoke tobacco products daily?
    - Yes
    - No
  - If yes, on average, how many times a day do you use such a product?
    - Once
    - Twice
    - Three times
    - Four times
    - >4 times
    - Unknown
  - How old were you when you first started smoking daily?
    Enter age in years…………………….
  - Do you currently use any smokeless tobacco such as [snuff, chewing tobacco, betel, e‐cigarettes]?
    - Yes
    - No
  - If yes, on average, how many times a day do you use such a product?
    - Once
    - Twice
    - Three times
    - Four times
    - >4 times
    - Unknown
  - In the past, did you ever smoke daily?
    - Yes
    - No
  - How old were you when you stopped smoking daily?
    Enter age in years…………………….
9 **Alcohol consumption**
  - Have you ever consumed an alcoholic drink such as beer, wine, spirits, fermented cider or [add other local examples e.g. palm wine]?
    - Yes
    - No
  - Have you consumed an alcoholic drink within the past 12 months?
    - Yes
    - No
  - During the past 12 months, how frequently have you had at least one alcoholic drink?
    - Daily.
    - 5–6 days per week
    - 3–4 days per week
    - 1–2 days per week
    - 1–3 days per month
  - Have you consumed an alcoholic drink within the past 30 days?
    - Yes
    - No
  - If yes, during the past 30 days, how many times did you have the following number of standard alcoholic drinks in a single drinking occasion?
    - Once
    - Twice
    - Three times
    - Four times
    - Five times
    - Six times
    - >Six times
10 **Diet**
  - In a typical week, on how many days do you eat fruit?
    - None
    - 1–2
    - 3–4
    - 5–6
    - Daily
  - How many servings of fruit do you eat on one of those days?
    - 1–2
    - 3–4
    - 5–6
  - In a typical week, on how many days do you eat vegetables?
    - None
    - 1–2
    - 3–4
    - 5–6.
    - Daily
  - How many servings of vegetables do you eat on one of those days?
    - 1–2
    - 3–4
    - 5–6
  - What type of oil or fat is most often used for meal preparation in your household?
    - Vegetable oil
    - Groundnut oil
    - Lard
    - Margarine or cooking butter
    - Palm oil
    - Don't know
    - Not applicable
      **Dietary salt**
  - How often do you add salt or a salty sauce, such as soy or Maggi sauce, to your food right before you eat it or as you are eating it?
    - Always
    - Often
    - Sometimes
    - Rarely
    - Never
    - Don't know
  - How often is salt, salty seasoning or a salty sauce added in cooking or preparing foods in your household?
    - Always
    - Often
    - Sometimes
    - Rarely
    - Never
    - Don't know
  - How often do you eat processed food high in salt? By processed food high in salt, I mean foods that have been altered from their natural state, such as packaged salty snacks and canned salty food, including pickles and preserves, salty food prepared at a fast food restaurant, cheese, bacon and processed meat [corned beef, etc.]
    - Always
    - Often
    - Sometimes
    - Rarely
    - Never
    - Don't know
  - How much salt or salty sauce do you think you consume?
    - Far too much
    - Too much
    - Just the right amount
    - Too little
    - Far too little
    - Don't know
11 **Physical activity**
  - Does your work involve vigorous‐intensity activity that causes large increases in breathing, carrying or lifting heavy loads, digging, or construction work for at least 10 min continuously?
    - Yes
    - No
  - In a typical week, on how many days do you do vigorous‐intensity activities as part of your work?
    - 0
    - 1
    - 2
    - 3
    - 4
    - 5
    - 6
    - 7
  - Does your work involve moderate‐intensity activity that causes small increases in breathing or heart rate, such as brisk walking [or carrying light loads] for at least 10 min continuously?
    - Yes
    - No
  - In a typical week, on how many days do you do moderate‐intensity activities as part of your work?
    - 0
    - 1
    - 2
    - 3
    - 4
    - 5
    - 6
    - 7
  - Do you do any vigorous‐intensity sports, fitness or recreational (leisure) activities that cause large increases in breathing or heart rate like running or football for at least 10 min continuously?
    - Yes
    - No
  - In a typical week, on how many days do you do vigorous‐intensity sports, fitness or recreational (leisure) activities?
    - 0
    - 1
    - 2
    - 3
    - 4
    - 5
    - 6
    - 7
  - How much time do you spend doing vigorous‐intensity sports, fitness or recreational activities on a typical day?
    - <1 h
    - 1–5 h
    - 6–10 h
    - >10 h
  - How much time do you usually spend sitting or reclining on a typical day?
    - <1 h>
    - 1–5 h
    - 6–10 h
    - >10 h
    - Not applicable
12 **History of diabetes**
  i Have you ever had your blood sugar measured by a doctor or other health worker? Yes 1 No 2
  j Have you ever been told by a doctor or other health worker that you have raised blood sugar or diabetes? Yes 1 No 2
  k Were you first told in the past 12 months? Yes 1 No 2
  l In the past 2 weeks, have you taken any drugs (medication) for diabetes prescribed by a doctor or other health worker? Yes 1 No 2
  m Are you currently taking insulin for diabetes, prescribed by a doctor or other health worker? Yes 1 No 2
  n Have you ever seen a traditional healer for diabetes or raised blood sugar? Yes 1 No 2
  o Are you currently taking any herbal or traditional remedies for your diabetes? Yes 1 No 2

**Section D: Disease characteristics**

13 **Date of diagnosis** (MM/YYYY): …………….
14 **Age at diagnosis** (years): ………….
15 **Date of first presentation in Diabetic Clinic** (mm/year) ………….
16 **Family history of diabetes mellitus?**
  - Yes
  - No
17 **If the answer to Q16 is yes, specify** (Circle all that apply)

- Father
- Mother
- Brother
- Sister
- Uncle
- Aunt
- Cousin
- Nephew
- Niece
- Grand father
- Grandmother

18 **Sibling pair status**

- Are you paternally related? Yes/No
- Are you maternally related? Yes/no
- Are you full Siblings Yes/no
- Are you half siblings? Yes/no

**Section E: Complications/co‐morbidities**

19 **Presence of complications/co‐morbidities?**
  - Yes
  - No
20 **If Yes, select type of complications/comorbidities: (select** as many as appropriate)
  - Hypertension
  - Heart disease/chest pain/ECG abnormalities/echo abnormalities
  - Dyslipidaemia
  - Obesity
  - Stroke
  - Retinopathy
  - Cataracts
  - Glaucoma
  - Blindness
  - Neuropathy
  - Foot ulcer (previous or current history)
  - Sexual dysfunction
  - Kidney disease
  - Limb amputation
21 **Year of diagnosis of complication** (YYYY): …………………

**Section F: Treatment plan**

22 **Type of treatment**
  - Diet and exercise alone
  - Diet, exercise and conventional medication
  - Traditional herbal remedies
  - Not on treatment
23 **Current diabetic medication patient is using** (Circle as many as appropriate)
  - Sulphonylurea (e.g. Glibenclamide, Gliclazide, Glipizide, Glimepiride)
  - Biguanide (Metformin)
  - DPP 4 Inhibitors (Vildagliptin, Sitagliptin, Linagliptin)
  - Alpha‐glucosidase inhibitors (acarbose, voglibose)
  - GLP‐1 receptor agonist (Liraglutide)
  - SGLT‐2 Inhibitor (Empagliflozin, Dapagliflozin, etc.)
  - Insulin
  - Others (please specify)
24 **Other medications currently being used by the patient** (Circle as many as appropriate)
  - ACEI (Lisinopril, Perindopril, Ramipril, enalapril, captop [ril.])
  - ARB (Valsartan, Losartan, Irbesartan, telmisartan, candesartan, omelsartan)
  - Diuretic (Indapamide, Hydrochlorothiazide, frusemide, torsemide, aldactone, amiloride, metolazone, eplerenone, chlothalidone)
  - Betablocker (bisoprolol, carvedilol, metoprol, labetalol)
  - Calcium channel blockers (amlodipine, nifedipine, felodipine, verapamil)
  - Alphablocker (doxasoxin, prazosin, tamsulosin, alfuzosin, terazosin)
  - Antiplatelet (aspirin, clopidogrel, prasugrel, ticagrelor.)
  - HMG‐coA reductase inhibitors (atorvastatin, simvastatin, rosuvastatin, pravastatin)
  - Phosphodiesterase inhibitors (tadalafil, sildenafil, etc.)
  - Others (pregabalin, gabapentin, etc.): Please specify _______________________.
25 **Blood pressure**
  Cuff size used: Small 1, Medium 2, Large 3
  Reading 1 Systolic (mmHg) └─┴─┴─┘ Diastolic (mmHg) └─┴─┴─┘
  Reading 2 Systolic (mmHg) └─┴─┴─┘ Diastolic (mmHg) └─┴─┴─┘
  Reading 3 Systolic (mmHg) └─┴─┴─┘ Diastolic (mmHg) └─┴─┴─┘
  During the past 2 weeks, have you been treated for raised blood pressure with drugs (medication) prescribed by a doctor or?
  Other health worker? Yes 1 No 2
26 **Height and weight**
  For women: Are you pregnant? Yes 1 No 2
  Height in centimetres (cm) └─┴─┴─┘ └─┘
  Weight kilogram (kg) └─┴─┴─┘.└─┘
27 **Waist**
  Waist circumference in centimetres (cm) └─┴─┴─┘.└─┘
28 **Hip circumference**
  Hip circumference in centimetres (cm) └─┴─┴─┘.└─┘
29 **Neck circumference**
  Male …………………….
  Female……………………
30 **Heart rate**
  Reading 1 Beats per minute └─┴─┴─┘
  Reading 2 Beats per minute └─┴─┴─┘
  Reading 3 Beats per minute └─┴─┴─┘
31 **Blood glucose**
  During the past 12 h, have you had anything to eat or drink, other than water? Yes 1 No 2
  Time of day blood specimen taken (24 h clock) Hours: minutes └─┴─┘: └─┴─┘ h min
  Fasting blood glucose
  [CHOOSE ACCORDINGLY: MMOL/L OR MG/DL] mmol/L └─┴─┘. └─┴─┘ mg/dL └─┴─┴─┘.└─┘
  Today, have you taken insulin or other drugs (medication) that have been prescribed by a doctor or other health worker for raised blood glucose? Yes 1 No 2
32 **Blood lipids**
  Total cholesterol
  [CHOOSE ACCORDINGLY: MMOL/L OR MG/DL] mmol/L └─┴─┘. └─┴─┘ mg/dL └─┴─┴─┘.└─┘
  **Triglycerides and HDL cholesterol**
  Triglycerides
  [CHOOSE ACCORDINGLY: MMOL/L OR MG/DL] mmol/L └─┴─┘. └─┴─┘mg/dL └─┴─┴─┘.└─┘
  HDL Cholesterol
  [CHOOSE ACCORDINGLY: MMOL/L OR MG/DL] mmol/L └─┘. └─┴─┘ mg/dL └─┴─┴─┘.└─┘
  During the past two weeks, have you been treated for raised cholesterol with drugs (medication) prescribed by a doctor or other health worker? Yes 1 No 2
33 **Other laboratory markers**
  Sex Hormone Binding Globulin
  C‐reactive protein…………
  Genotype…………………
  Haemoglobin levels………….
  Iron levels………………
  Folate levels…………………
  Vitamin D…………………….
  Calcium level……………………
34 **Urinary albumin creatinine ratio (uACER)**
  Had you been fasting prior to the urine collection? Yes 1 No 2
  Urinary albumin mmol/L └─┴─┴─┘.└─┘
  Urinary creatinine mmol/L └─┴─┘ └─┴─┘
  UACER………………
